# Enhancement of 1,3-propanediol production from industrial by-product by *Lactobacillus reuteri* CH53

**DOI:** 10.1186/s12934-019-1275-x

**Published:** 2020-01-13

**Authors:** Jung-Hyun Ju, Dexin Wang, Sun-Yeon Heo, Min-Soo Kim, Jeong-Woo Seo, Young-Min Kim, Dae-Hyuk Kim, Soon-Ah Kang, Chul-Ho Kim, Baek-Rock Oh

**Affiliations:** 1 Microbial Biotechnology Research Center, Jeonbuk Branch Institute, Korea Research Institute of Bioscience and Biotechnology (KRIBB), Jeongeup, Jeonbuk 56212 Republic of Korea; 2 Department of Food Science & Technology and Functional Food Research Center, Chonnam National University, Gwangju, 61186 Republic of Korea; 3 Radiation Utilization and Facilities Management Division, Korea Atomic Energy Research Institute, Jeongeup, Jeonbuk 56212 Republic of Korea; 4 Institute for Molecular Biology and Genetics, Center for Fungal Pathogenesis, Chonbuk National University, Jeonju, Jeonbuk 54896 Republic of Korea; 5 Department of Conversing Technology, Graduate School of Venture, Hoseo University, Seoul, 06724 Republic of Korea

**Keywords:** Biorefinery, 1,3-propandiol, *Lactobacillus reuteri*, Crude glycerol, Corn steep liquor

## Abstract

**Background:**

1,3-propanediol (1,3-PDO) is the most widely studied value-added product that can be produced by feeding glycerol to bacteria, including *Lactobacillus* sp. However, previous research reported that *L*. *reuteri* only produced small amounts and had low productivity of 1,3-PDO. It is urgent to develop procedures that improve the production and productivity of 1,3-PDO.

**Results:**

We identified a novel *L*. *reuteri* CH53 isolate that efficiently converted glycerol into 1,3-PDO, and performed batch co-fermentation with glycerol and glucose to evaluate its production of 1,3-PDO and other products. We optimized the fermentation conditions and nitrogen sources to increase the productivity. Fed-batch fermentation using corn steep liquor (CSL) as a replacement for beef extract led to 1,3-PDO production (68.32 ± 0.84 g/L) and productivity (1.27 ± 0.02 g/L/h) at optimized conditions (unaerated and 100 rpm). When CSL was used as an alternative nitrogen source, the activity of the vitamin B12-dependent glycerol dehydratase (*dhaB*) and 1,3-propanediol oxidoreductase (*dhaT*) increased. Also, the productivity and yield of 1,3-PDO increased as well. These results showed the highest productivity in *Lactobacillus* species. In addition, hurdle to 1,3-PDO production in this strain were identified via analysis of the half-maximal inhibitory concentration for growth (IC50) of numerous substrates and metabolites.

**Conclusions:**

We used CSL as a low-cost nitrogen source to replace beef extract for 1,3-PDO production in *L. reuteri* CH53. These cells efficiently utilized crude glycerol and CSL to produce 1,3-PDO. This strain has great promise for the production of 1,3-PDO because it is generally recognized as safe (GRAS) and non-pathogenic. Also, this strain has high productivity and high conversion yield.

## Background

1,3-Propanediol (1,3-PDO, CH_2_CH_2_(OH)_2_) is a viscous liquid that is miscible with water, and an important intermediary used for the production of polymers from petrochemical compounds. It is mainly used for production of the polyester polytrimethylene terephthalate (PTT) [1, 2], but also for the manufacture of other polymers, cosmetics, foods, lubricants, and medical products [3].

Chemical synthesis of 1,3-PDO is by the hydration of acrolein, or the hydroformylation of ethylene oxide to 3-hydroxypropionaldehyde, followed by hydrogenation [4]. The current demand for biofuels and biopolymers is driving research to increase the production efficiency and reduce costs. Biotechnology has environmental and economic advantages for biofuel production, because it allows use of renewable materials for the synthesis of 1,3-PDO. Some species in the bacterial genera *Klebsiella* [5–7], *Citrobacter* [8], *Clostridium* [9, 10], and *Lactobacillus* [11–13] can naturally convert glycerol into 1,3-PDO. With glycerol as a carbon source, a mutant of *Klebsiella pneumoniae* can produce 102.7 g/L 1,3-PDO [7] and *Clostridium butyricum* can produce up to 94 g/L 1,3-PDO [10]. DuPont and Genencor International used a recombinant *E. coli* to produce up to 135 g/L 1,3-PDO from glucose [14]. However, use of these strains is problematic because of pathogenicity, the need for anaerobic growth, and the need for genetic recombination. Thus, it is imperative to select non-pathogenic, non-recombinant, and environmentally friendly strains for the commercial production of 1,3-PDO.

*Lactobacillus reuteri* is a hetero-fermentative bacterium that inhabits the gastrointestinal tracts of humans, pigs, birds, and other animals. This microorganism can produce 3-HPA (3-hydroxypropionaldehyde) and 1,3-PDO, is “generally recognized as safe” (GRAS), non-pathogenic, and not genetically engineered. A disadvantage is that *L*. *reuteri* cannot grow on glycerol as the sole carbon source, so there is a need for co-fermentation (e.g., a mixture of glycerol and glucose in the culture medium) to produce 1,3-PDO. Talarico et al. [15] characterized the carbohydrate metabolism of *L*. *reuteri*. Their results showed that sugar fermentation results in the production of lactate, CO_2_, acetate, and ethanol when glucose is the electron acceptor. These cells, and bacterial cells generally, regenerate NADH during lactate and ethanol synthesis, and produce ATP during acetate synthesis. In addition, glycerol dehydratase (which requires a vitamin B_12_ cofactor) converts glycerol into 3-HPA, which is subsequently reduced by 1,3-propanediol oxidoreductase (an NAD^+^-dependent oxidoreductase) into 1,3-PDO. Since 1990, when Talarico et al. reported that *L*. *reuteri* produces small amounts of 1,3-PDO [15], other researchers have examined 1,3-PDO production in other species of *Lactobacillus* [11–13, 16–18]. However, all other tested *Lactobacillus* species only have low productivity of this compound.

Glycerol is an essential carbon source for the production of 1,3-PDO from lactic acid bacteria. Crude glycerol is a by-product of the biodiesel industry, and about 10% (w/w) glycerol is produced during biodiesel production [19]. The glycerol produced from a biodiesel plant is usually 40–70% (w/w) before acid treatment, and 80% (w/w) after acid treatment [20]. The amount of industrial crude glycerol production has increased as the biodiesel industry has grown. To improve the economic competitiveness of biodiesel production, it is therefore imperative to develop sustainable production of crude glycerol. This has motivated many studies to examine the production of a high-value product from crude glycerol using *Lactobacillus* [12, 21–23].

Nitrogen also plays an important role in microbial fermentation, and a cheap and simple organic nitrogen compound is preferable to expensive and complex nitrogen sources, such as yeast extract and beef extract [24]. Corn steep liquor (CSL), a by-product of the starch industry, is an inexpensive nitrogen source that can be used for cultivation of microorganisms, because it contains a rich complement of nitrogenous compounds, vitamins, amino acids, and biotins [25, 26].

The aim of the present study is to examine the effect of various operational strategies (i.e., different levels of aeration and agitation, and different nitrogen sources) on the production and productivity of 1,3-PDO using the newly isolated *L*. *reuteri* CH53. In particular, we examined 1,3-PDO production using crude glycerol (a by-product of biodiesel production) and using CSL as a low-cost nitrogen source. Also, we examined the potential for further improvements in 1,3-PDO production by IC50 analysis of numerous substrates and metabolites.

## Methods

### Chemicals and media

Crude glycerol (80% purity, percent weight per weight) and CSL were purchased from GS Bio (Yeosu, Korea) and Samyang Genex (Incheon, Korea), respectively. All other chemicals were of analytical grade. De Man, Rogosa, Sharpe (MRS) medium with crude glycerol was used for pre-culturing and 1,3-PDO production. Each liter of MRS medium contained 10 g peptone, 10 g beef extract, 5 g yeast extract, 1 g Tween-80, 2 g K_2_HPO_4_, 2 g ammonium citrate, 5 g sodium acetate, 0.2 g MgSO_4_, and 0.05 g MnSO_4_. The concentrations of glucose and crude glycerol were varied.

### Isolation of *L*. *reuteri* CH53

Porcine small intestine and duodenum samples were collected from Woori Bio-Food & Bio-Tech Co., Ltd. (Iksan, Korea). First, 1 g of porcine small intestine and duodenum samples were serially diluted in sterile PBS buffer (pH 6.8). Then, 100 µL samples were smeared onto the surface of MRS agar (Difco, USA) containing 0.3 g/L bromocresol purple (Sigma, USA). The Petri dishes were then incubated at 37 °C for 24 h in a Bactron Anaerobic Chamber (Shellab, USA). The primary isolation was conducted using a colorimetric method using bromocresol purple. Final identification of strains was performed using the matrix-assisted laser desorption ionization (MALDI) Biotyper system (Bruker Daltonics, USA), with the modification described by Buchan et al. [27].

### Identification of *L*. *reuteri* CH53 by 16S rDNA amplification

Genomic DNA was obtained using a Genomic DNA Purification Kit from Invitrogen. The 16S rRNA gene was amplified by PCR using two universal primers, 27F (5′-AGAGTTTGATCMTGGCTCAG-3′) and 1492R (5′-GGTTACCTTGTTACGACTT-3′). The PCR conditions consisted of an initial denaturation step at 95 °C for 5 min; followed by 30 cycles at 95 °C for 30 s, 55 °C for 30 s, and 72 °C for 90 s; and a final step at 72 °C for 7 min. The nucleotide sequence was determined from the amplified PCR fragment by Solutions for Generic Technologies (Solgent, Korea) and compared with available 16S rRNA gene sequences from GenBank (www.ncbi.nlm.nih.gov/blast) and EzTaxon (eztaxon-e.ezbiocloud.net) [28]. Phylogenetic analysis was performed using MEGA6 [29] with the neighbor-joining method [30]. Bootstrap values were calculated based on 1000 replicates [31].

### Fermentation of *L*. *reuteri* CH53

Seed cells for fermentation were prepared in 500 mL round flasks containing 300 mL of MRS medium with glycerol. Flasks were static-incubated at 37 °C for about 8 h, and cultures were subsequently inoculated into growth vessels at a concentration of 10% (v/v). Batch and fed-batch fermentations were conducted in a 5 L stirred-vessel system (Kobiotech. Co. Ltd., Incheon, Korea) that contained 3 L of MRS medium with glycerol. Unless otherwise stated, all fermentation experiments were conducted at 37 °C without aeration (only agitation, no sparging of air or N_2_ gas). The effect of agitation speed was determined by growing cells at 50, 100, 200, and 300 rpm. The feed to the fed-batch fermentation was using a feeding solution containing 450 g of glucose and 450 g of glycerol in 1 L distilled water, and the molar ratio of glucose-to-glycerol was 0.5. The glucose and glycerol feeding began after glucose was completely consumed at a constant feed rate of 21 mL/h (from 4 to 21 h). Unless otherwise stated, the pH was maintained at pH 5.5 ± 0.2 using 28% (w/v) NH_4_OH or 2 M HCl. All presented results are averages from three independent experiments. Cell growth was monitored by removing aliquots at various times and measurement of OD_600nm_ using a UV–Vis spectrophotometer (Ultrospec 3100 Pro; Amersham Biosciences, Piscataway, NJ, USA). Cells were used for enzyme activity assays, and culture broth was analyzed for metabolites.

### Metabolites and total nitrogen analysis

Metabolites and glucose in the culture broth were determined using a high-performance liquid chromatography (HPLC) system (Agilent 1200) that was equipped with a refractive index detector (RID) and an Aminex HPX-87H column (300 × 78 mm; Bio-Rad, Hercules, CA, USA). The mobile phase was 2.5 mM H_2_SO_4_, and the flow rate was 0.6 mL/min. The column and cell temperatures were 65 °C and 45 °C, respectively [32]. Total nitrogen concentration was measured using a HS-TN (CA)-L kit (concentration range: 1 to 50 mg/L; Humas, Korea) with a HS-2300 plus water analyzer.

### Enzyme activity analysis

*Lactobacillus*. *reuteri* CH53 cells were harvested by centrifugation and washed two times with 100 mM phosphate buffer (pH 7.0). Cells in the same buffer were disrupted using an ultrasonic system (crushing 2 s and rest 6 s for 12 min, Power: 30%, 210 W, 19,736 Hz). A crude extract was obtained by centrifugation for 10 min at 13,000 rpm, and protein concentration was determined by the Bradford assay, using bovine serum albumin (BSA) as a standard. The activity of the vitamin B_12_-dependent glycerol dehydratase (*dhaB*) was determined by measuring acrolein absorbance at 560 nm [33]. Because 1 mol of 3-HPA is dehydrated with 1 mol of acrolein, the data were simply expressed as 3-HPA concentration, and 1 U of enzyme activity was defined as the amount of enzyme required to form 1 mmol of 3-HPA per min. The activity of 1,3-propanediol oxidoreductase (*dhaT*) was determined by measuring NADH absorbance at 340 nm (ε_NADH_ = 6220 L/mol/cm) [16], in which one unit of enzyme activity (U) corresponds to the generation of 1 μmol of NADH per min. Specific activity is expressed U/mg protein.

### Measurement of IC50

A 96-multiwell plate-based assay was used to assess the effect of each compound (glucose, glycerol, lactic acid, acetic acid, ethanol and 1,3-PDO) on 1,3-PDO production. Seed cells from an overnight culture were washed with sterile PBS (pH 5.5), and then 1% (v/v) aliquots were inoculated into an MRS-based medium containing different concentrations of the different compounds. Then 300 μL of the inoculated medium was added to each well and incubated at 37 °C in a Bactron Anaerobic Chamber (Shellab, USA). To measure bacterial growth, OD was monitored at a wavelength of 600 nm every 2 h for 12 h using a 96-well Microplate Reader (BioTek, Korea). To calculate the IC50 for each compound, the biomass (OD600_nm_) after 10 h was plotted against the log_10_ of the concentration.

## Results and discussion

### Isolation and identification *L*. *reuteri* CH53

**Fig. 1:**
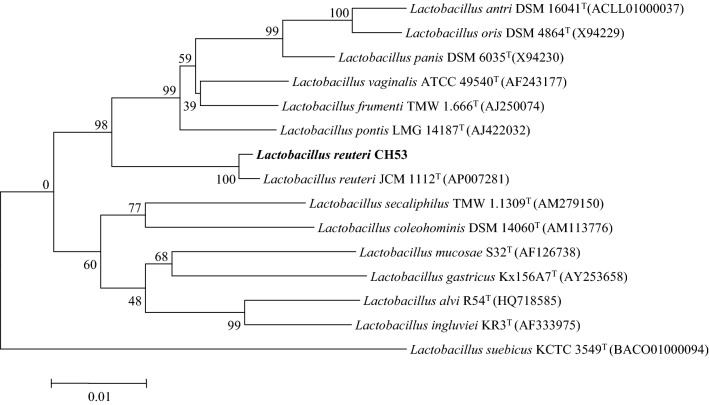
Relationship of *L*. *reuteri* CH53 with other *Lactobacillus* strains. The neighbor-joining phylogenetic tree was based on 16S rRNA gene sequences, and the number at each internal branch shows the bootstrap value (%) for the node calculated from 1000 replicates

We identified several *Lactobacillus* isolates from the porcine small intestine and duodenum samples (based on their brown color on MRS agar plates), and tested them for growth and 1,3-PDO production in MRS medium using glycerol and glucose as co-substrates under anaerobic conditions. One isolate, *L*. *reuteri* CH53, produced abundant 1,3-PDO and grew faster than all other isolates. We investigated the identity this isolate using 16S rDNA analysis. Analysis using NCBI BLAST showed it had high similarity (> 99%) to *Lactobacillus reuteri* JCM 1112T (AP007281). Thus, we entered the 16S rDNA sequence into the NCBI nucleotide sequence database (Accession No. MF461465) and used the neighbor-joining method to determine its relationship to other strains of *Lactobacillus* (Fig. 1). The results indicated that *L*. *reuteri* CH53 grouped with other strains in the genus *Lactobacillus*, and most closely with *L*. *reuteri* JCM1112 (bootstrap value: 100%). This 16S rRNA phylogenetic tree thus indicates that *L*. *reuteri* CH53 was in a well-supported monophyletic group that includes other *Lactobacillus* strains.

### Effect of aeration on 1,3-PDO production in glucose–glycerol batch fermentation

We examined the effect of aeration by batch-fermentation of *L*. *reuteri* CH53 under aerobic condition (sparging: 1.0 volume of air per volume of liquid per min [vvm]), unaerated condition (no sparging of air), and anaerobic condition (sparging: N_2_ 1.0 vvm) at 200 rpm. These experiments used MRS medium with 20 g/L glucose and 25 g/L crude glycerol in a 5 L bioreactor with a 3 L working volume. Table 1 shows the results from these three batch-fermentation conditions at 10 h. When the growth medium was aerated, 1,3-PDO production and cell growth were remarkably lower than under the other two conditions. The initial glycerol was almost all consumed under unaerated and anaerobic conditions, but 17.38 ± 0.31 g/L of the glycerol remained under aerobic conditions. Fermentation under unaerated and anaerobic conditions led to production of 11.57 ± 0.24 g/L and 11.88 ± 0.12 g/L 1,3-PDO, respectively. These results thus indicate that aerobic conditions are not suitable for cell growth and 1,3-PDO production in this strain.

**Table 1:** Effect of different aeration conditions on 1,3-propanediol production in batch fermentation of *L*. *reuteri* CH53 using glucose and glycerol as co-substrates

Aeration condition	Biomass (OD_600_)	1,3-PDO (g/L)	Acetate (g/L)	Lactate (g/L)	Y_1,3-PDO/gly_ g/g)	Q_1,3-PDO_ (g/L/h)	Residual glycerol (g/L)
Aerobic (air: 1.0 vvm)	2.03 ± 0.14	1.51 ± 0.09	0.27 ± 0.03	3.71 ± 0.16	0.55 ± 0.08	0.15 ± 0.01	17.38 ± 0.31
Anaerobic (N_2_: 1.0 vvm)	7.02 ± 0.24	11.88 ± 0.12	3.82 ± 0.11	10.99 ± 0.85	0.60 ± 0.01	1.19 ± 0.01	0.27 ± 0.18
Unaerated	6.98 ± 0.38	11.57 ± 0.24	3.79 ± 0.13	10.94 ± 0.73	0.61 ± 0.02	1.16 ± 0.02	1.07 ± 0.13

Our finding of no significant differences in production of 1,3-PDO under unaerated and anaerobic conditions may be because lactic acid bacteria maintain anaerobic conditions inside the incubator due to their generation of CO_2_. In agreement, previous research reported that *L*. *reuteri* ATCC 55730 converted glycerol to 1,3-PDO more efficiently under unaerated and anaerobic conditions than under aerated conditions [11]. Geueke et al. [34] reported that increased oxygen promotes the activity of NADH oxidase, which regenerates NAD^+^ and results in low production of 1,3-PDO. From an effective production point of view, unaerated conditions are also more desirable for 1,3-PDO production because many restrictions place limits on the supply of N_2_. Thus, we used unaerated conditions to measure 1,3-PDO production by *L*. *reuteri* CH53 in all subsequent experiments.

### Effect of agitation speed on production of 1,3-PDO

We tested the effect of agitation speed on 1,3-PDO production using 8 h batch-fermentation in MRS medium with 20 g/L glucose and 25 g/L crude glycerol without aeration (Table 2). The results indicated that cell growth decreased as agitation speed increased above 100 rpm. Maximal production (15.58 ± 0.56 g/L) and productivity (1.95 ± 0.07 g/L/h) of 1,3-PDO occurred at an agitation speed of 100 rpm. These conditions are similar to those used for cultivation of other *Lactobacillus* strains [17].

**Table 2:** Effect of agitation speed on 1,3-propanediol production in batch fermentation of *L*. *reuteri* CH53 using glucose and glycerol as co-substrates

Agitation speed (rpm)	Biomass (OD_600_)	1,3-PDO (g/L)	Acetate (g/L)	Lactate (g/L)	Y_1,3-PDO/gly_ (g/g)	Q_1,3-PDO_ (g/L/h)	Residual glycerol (g/L)	Residual glucose (g/L)
50	8.40 ± 0.26	14.79 ± 0.38	7.09 ± 0.26	13.27 ± 0.39	0.73 ± 0.02	1.83 ± 0.05	0.00	0.00
100	8.35 ± 0.24	15.58 ± 0.56	4.77 ± 0.18	14.18 ± 0.38	0.78 ± 0.03	1.95 ± 0.07	0.00	0.00
200	6.71 ± 0.18	10.75 ± 0.42	4.10 ± 0.16	10.18 ± 0.24	0.72 ± 0.01	1.34 ± 0.05	2.02 ± 0.48	1.42 ± 0.26
300	6.20 ± 0.09	7.57 ± 0.31	3.05 ± 0.09	7.73 ± 0.22	0.71 ± 0.06	0.95 ± 0.04	9.35 ± 0.56	4.02 ± 0.35

There are no previous reports on the effect of agitation speed on glycerol-glucose based 1,3-PDO production by other *Lactobacillus* strains. As the agitation speed increases, the culture broth strikes the baffle of fermentor, and this increases the surface area in contact with air, thus exposing the cells to more oxygen [35]. As reported above, we found that aerobic conditions are not suitable for cell growth and 1,3-PDO production. Thus, it is important to uniformly distribute the cells in the medium through proper agitation but without excessive splashing for efficient production of 1,3-PDO. We used an agitation speed 100 rpm for all subsequent experiments.

### Effect of fed-batch fermentation on production of 1,3-PDO

**Fig. 2:**
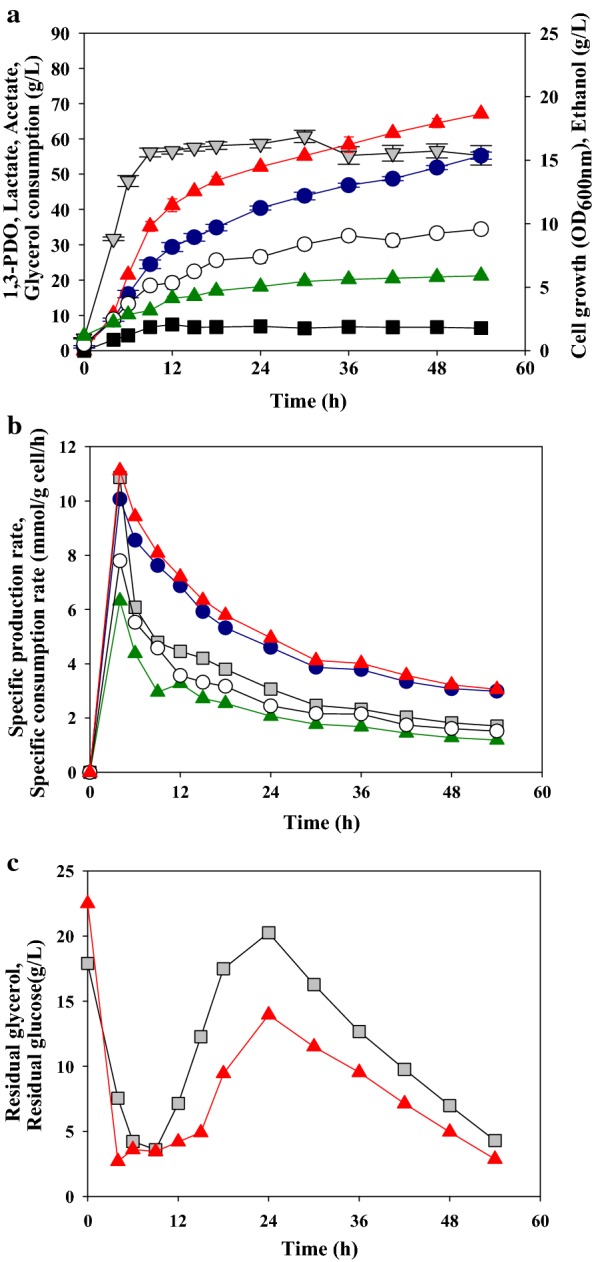
Production of 1,3-propanediol and fermentation profile during fed-batch fermentation of *L*. *reuteri* CH53. **a** Gray down-triangles, cell growth; red up-triangles, glycerol consumption; blue circles, 1,3-propanediol; empty circles, lactic acid; green up-triangles, acetic acid; black squares, ethanol. **b** Gray squares, glucose; red up-triangles, glycerol; blue circles, 1,3-propanediol; empty circles, lactic acid; green up-triangles, acetic acid. **c** Gray squares, glucose; red up-triangles, glycerol

Efficient fed-batch fermentation can increase the production of target bio-products [36, 37]. Thus, we tested the effect of fed-batch fermentation on 1,3-PDO production by *L*. *reuteri* CH53 under optimized conditions (unaerated and 100 rpm) (Fig. 2). The molar ratios of the feeding solution for efficient fed-batch fermentation indicated the optimal molar ratio of glucose to glycerol was 0.5 (data not shown). During fed-batch fermentation, despite the presence of sufficient glucose in the culture broth, cell growth entered a stationary phase after 9 h, which is thought to be due to the depletion of substrates essential for cell division in addition to glucose. After cell growth stopped, glucose consumption rate decreased, carbon sources accumulated in the culture medium. The concentration of glucose and glycerol in the culture broth decreased slowly after feeding stopped. The specific production rate and the specific consumption rate showed the highest result at 4 h. The specific production rate and the specific consumption rate were decreased rapidly due to cell growth up to 9 h. After the growth of cells ceased, the specific production rate and the specific consumption rate gradually decreased, as production rate and consumption rate decreased. Similar to the consumption of glucose, the consumption rate of glycerol was reduced and the production rate of 1,3-PDO was also reduced. *L*. *reuteri* strain have separate glucose and glycerol metabolic pathways, respectively [15]. The production of 1,3-PDO is converted from glycerol, and that requires NADH produced by glucose metabolism. The decrease in glucose consumption rate led to a decrease in NADH production, which led to a decrease in glycerol consumption and 1,3-PDO production. The maximum production of 1,3-PDO through fed-batch fermentation was 55.24 ± 1.02 g/L at 54 h and the productivity was 1.02 ± 0.02 g/L/h. The total amount of consumed glycerol was 67.36 ± 0.53 g/L. The maximum theoretical conversion yield of 1,3-PDO from glycerol is 0.83 g 1,3-PDO per g glycerol (mol-to-mol conversion) [38]. Our conversion yield was 0.82 g 1,3-PDO per g glycerol, 98.8% of theoretical maximum. A high conversion yield is important for the economical production of 1,3-PDO in bio-refinery processes [39]. Our results thus indicate that *L*. *reuteri* CH53 has potential as an efficient 1,3-PDO producer.

### Effect of 1,3-PDO production using CSL as a nitrogen source

**Fig. 3:**
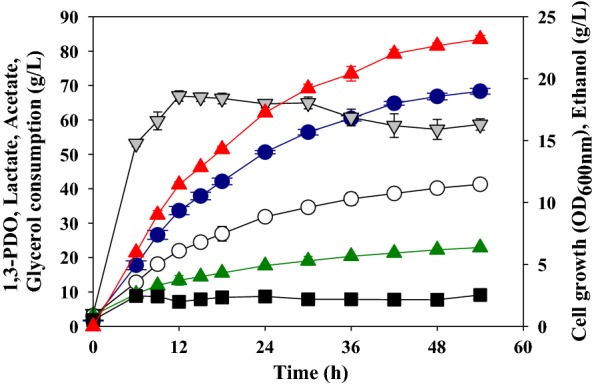
Production of 1,3-propanediol and other products during fed-batch fermentation of *L*. *reuteri* CH53 using CSL as the carbon source. Gray down-triangles, cell growth; red up-triangles, glycerol consumption; blue circles, 1,3-propanediol; empty circles, lactic acid; green up-triangles, acetic acid; black squares, ethanol

In general, the fermentation of lactic acid bacteria is performed using MRS medium, and beef extract is the most expensive component of this medium. Thus, we tested the effect of using untreated CSL as an alternative nitrogen source on 1,3-PDO production by *L*. *reuteri* CH53. Beef extract contained 11.4% total nitrogen, and CSL contained 4.1% total nitrogen (Table 3), so 30 g/L CSL used to replace 10 g/L beef extract. The fed-batch fermentation efficiency of 1,3-PDO production improved significantly when CSL was used as a replacement (Fig. 3 and Table 4). In particular, CSL increased cell growth and led to greater production of 1,3-PDO in a shorter time than beef extract. When cells were grown for 54 h in fed-batch fermentation with CSL, the maximum 1,3-PDO production was 68.32 ± 0.84 g/L and productivity was 1.27 ± 0.02 g/L/h, the lactate production was 41.27 ± 0.78 g/L, and the acetate production was 22.92 ± 0.31 g/L. Thus, compared to using beef extract, use of CSL led to greater 1,3-PDO production (68.32 ± 0.84 g/L vs. 55.24 ± 1.02 g/L) and productivity (1.27 ± 0.02 g/L/h vs. 1.02 ± 0.02 g/L/h). The CSL used in the experiment contained 7.1% lactic acid and 0.9% glucose. However, the 30 g/L CSL used in the fed-batch fermentation contains only 2.1 g/L lactic acid and 0.3 g/L glucose. There was no negative effect on fed-batch fermentation, such as inhibiting cell growth or suppressed 1,3-PDO production, because the concentration of lactic acid is very low compared to the IC50 value.

**Table 3:** Chemical characteristics of beef extract and CSL

	Beef extract	CSL
Total nitrogen (%)	11.4	4.1
Glucose (%)	–	0.9
Lactic acid (%)	–	7.1
pH	7.3	4.6

**Table 4:** Effect of using CSL and BE as substrates on production of 1,3-propanediol in fed-batch fermentation of *L*. *reuteri* CH53

Time (h)	Nitrogen source	Biomass (OD_600_)	1,3-PDO (g/L)	Acetic acid (g/L)	Lactic acid (g/L)	Q_1,3-PDO_ (g/L/h)
24	BE	16.28 ± 0.90	40.39 ± 0.59	15.35 ± 0.92	26.54 ± 1.06	1.68 ± 0.02
	CSL	17.96 ± 0.73	50.63 ± 0.77	17.65 ± 0.46	31.84 ± 0.88	2.11 ± 0.03
54	BE	15.36 ± 0.76	55.24 ± 1.02	21.23 ± 0.32	34.38 ± 0.78	0.99 ± 0.02
	CSL	15.36 ± 0.50	68.32 ± 0.84	22.92 ± 0.31	41.27 ± 0.78	1.27 ± 0.02

*BE* beef extract, *CSL* corn steep liquor

**Fig. 4:**
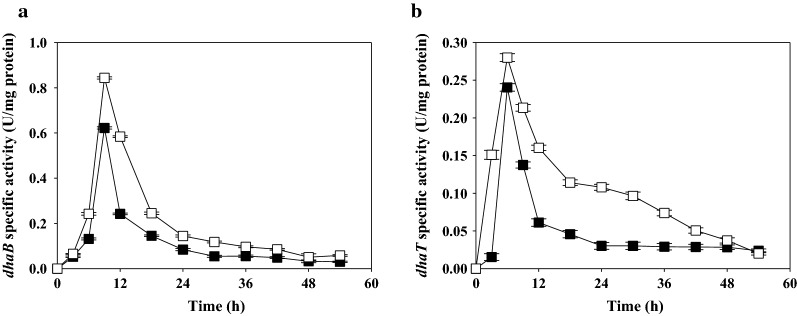
Effect of CSL and beef extract on changes in enzyme activity of *dhaB* (**a)** and *dhaT* (**b)** during fed-batch fermentation of *L*. *reuteri* CH53. Closed squares, beef extract; open squares, CSL

To examine the possible mechanism by which CSL improved the productivity of 1,3-PDO, we assayed enzyme activity during fed-batch fermentation (Fig. 4). The results show that cultures with 3% (w/v) CSL had much greater *dhaB* and *dhaT* activity than the controls up to 48 h. *dhaB* catalyzes the production of 3-HPA, a precursor of 1,3-PDO, and is a vitamin B12 dependent enzyme. It is thus possible that CSL increased *dhaB* enzyme activity because it supplies vitamin B12 and additional nutrients not supplied by beef extract. Similarly, Pflügl et al. [17], reported vitamin B12 addition significantly enhanced the production of 1,3-PDO by *Lactobacillus diolivorans* and Wischral et al. [40] reported enhanced production of 1,3-PDO by *Clostridium beijerinckii* when CSL was used as a nitrogen source. These results suggest that CSL can be used as an effective and low-cost nitrogen source for 1,3-PDO production by *L*. *reuteri* CH53, and is a suitable replacement for beef extract.

### 1,3-PDO production and productivity of other *Lactobacillus* strains

Previous studies have also examined 1,3-PDO production by other *Lactobacillus* strains (Table 5). The highest reported production of 1,3-PDO was 92 g/L at 165 h by *L*. *diolivorans* DSM 14421 [18], although the productivity from this strain was only about 0.56 g/L/h. Jolly et al. [11] reported a 1,3-PDO production of 65.3 g/L when *L*. *reuteri* ATCC 55730 was grown in glycerol, but the conversion yield was 0.80 g 1,3-PDO per g glycerol, and the productivity was only 0.47 g/L/h. Our strain produced 1,3-PDO of 68.32 g/L from fed-batch fermentation at 54 h, had a productivity of 1.27 g/L/h, and had a conversion yield near the theoretical maximum of 0.83 g 1,3-PDO per g glycerol. These results indicate that *L*. *reuteri* CH53 has the highest 1,3-PDO production and productivity among tested strains of *L*. *reuteri*.

**Table 5:** Fermentation of glycerol to 1,3-PDO by different *Lactobacillus* strains

Strain	1,3-PDO (g/L)	Yield (g/g)	Fermentation method	Productivity (g/L/h)	References
*L*. *reuteri* ATCC55730	65	0.80	Fed-batch	0.47	[11]
*L*. *diolivorans* DSM14421	85	0.53	Fed-batch	0.45	[12]
*L*. *reuteri* DSM20016	46	0.71	Fed-batch	0.92	[13]
*L*. *panis* PM1	16	0.72	Batch	0.08	[16]
*L*. *diolivorans* DSM14421	85	0.47	Fed-batch	0.60	[17]
*L*. *diolivorans* DSM14421	92	0.78	Fed-batch	0.56	[18]
*L*. *reuteri* CH53	68	0.82	Fed-batch	1.27	This study

However, *L*. *reuteri* CH53 performs worse than other *Lactobacillus* strains in terms of production concentration. We measured IC50 values to confirm the effect of different fermentation substrates and metabolites on cell growth and 1,3-PDO production (Table 6). IC50 is the concentration of agent that reduces cell growth by 50% under specified experimental conditions [13]. This allowed us to find the bottleneck of 1,3-PDO production. The IC50 values of glucose and glycerol were more than 1.5 M, so these substrates did not affect cell growth. The IC50 value of ethanol was 0.64 M (29.5 g/L), but these cells produced very little ethanol during fermentation, so ethanol did not have a significant effect. The IC50 of lactic acid was 0.31 M (27.9 g/L) and the IC50 of acetic acid was 0.29 M (17.4 g/L). When the 1,3-PDO concentration reached 50 g/L, the productivity was 2.1 g/L/h within the first 24 h (Table 4), and 1,3-PDO productivity and cell growth decreased rapidly after 24 h. At that time, the concentrations of lactic acid (31.8 g/L) and acetic acid (17.7 g/L) exceeded their IC50 values. Inhibition of cell growth by high lactic acid and acetic acid concentrations is caused by decreased co-factor production and enzyme activity, thus inhibiting 1,3-PDO production. In addition, the IC50 value of 1,3-PDO was 79.9 g/L, higher than the 68.3 g/L produced during the fed-batch fermentation. These results mean that *L*. *reuteri* CH53 can potentially produce more 1,3-PDO than described here, and that it is necessary to develop strains with enhanced resistance to lactic acid and acetic acid to further increase 1,3-PDO production. If a strain with enhanced organic acid resistance is developed using a traditional strain development methods (gamma-irradiation mutation, adaptive laboratory evolution, etc.), the advantage of using a GRAS can be maintained, and production of 1,3-PDO by *L*. *reuteri* CH53 can be competitive production.

**Table 6:** IC50 values of various substrates and metabolites in *L*. *reuteri* CH53

Compound	IC50 (M)
Glucose	1.73
Glycerol	1.52
1,3-PDO	1.05
Ethanol	0.64
Lactic acid	0.31
Acetic acid	0.29

## Conclusions

Previous studies have examined the production of 1,3-PDO by various lactic acid bacteria. These bacteria are useful 1,3-PDO producer because they are easy to culture and have the advantage of being GRAS. In this study, we optimized the culture of a new isolate, *L*. *reuteri* CH53, and improved the efficiency of 1,3-PDO production using crude glycerol and CSL as substrates. CSL was useful as an alternative nitrogen source, and it increased 1,3-PDO production by 1.24-fold compared to MRS medium. Our results suggest that fermentation of *L*. *reuteri* CH53 using CSL as alternative nitrogen source may provide more economical and efficiently production of 1,3-PDO.

## Data Availability

The datasets supporting the conclusions of this article are included within the article.

## Abbreviations

*L*. *reuteri*: *Lactobacillus reuteri*
1,3-PDO: 1,3-propanediol
GRAS: generally recognized as safe
GMO: genetically modified organism
3-HPA: 3-hydroxypropionaldehyde
DhaB: coenzyme B_12_-dependent glycerol dehydratase
DhaT: 1,3-PDO oxidoreductase
NAD^+^: dependent glycerol dehydrogenase
MRS: Deman, Rogosa, Sharpe
OD_600nm_: optical density _600nm_
CSL: corn steep liquor
BE: beef extract
IC50: the half maximal inhibitory concentration
